# Short- and Long-Range Dispersal by Members of the *Simulium damnosum* Complex (Diptera: Simuliidae), Vectors of Onchocerciasis: A Review

**DOI:** 10.3390/insects15080606

**Published:** 2024-08-12

**Authors:** Robert A. Cheke, Frances M. Hawkes, Manuela Carnaghi

**Affiliations:** Natural Resources Institute, University of Greenwich at Medway, Central Avenue, Chatham Maritime, Kent ME4 4TB, UK; f.m.hawkes@greenwich.ac.uk (F.M.H.); manuela.carnaghi@greenwich.ac.uk (M.C.)

**Keywords:** blackflies, *Simulium*, river blindness, onchocerciasis, migration, dispersal, flight distance

## Abstract

**Simple Summary:**

Blackflies are small blood-sucking flies that can carry and transmit the parasitic worm *Onchocerca volvulus*, the causative agent of human onchocerciasis, also known as “river blindness”. As gravid blackfly females oviposit their eggs in the fast-moving water of rivers and streams, the geographic distributions of both the vectors and the parasite that they transmit are closely linked with river basins. How blackflies disperse from and return to their breeding sites, the spatial distribution of their movements, and the distances that they can travel are crucial factors influencing transmission. As many endemic countries strive for elimination, understanding these patterns is important for guiding the design of monitoring and surveillance efforts. Here, we summarize the literature on the short-range and long-range dispersal of members of the *Simulium damnosum* complex and other onchocerciasis vectors and how this may influence exposure to blackfly bites, highlight significant gaps in existing knowledge, and propose possible directions for future research. This is particularly important in view of current global onchocerciasis elimination programs.

**Abstract:**

Blackfly members of the *Simulium damnosum* complex are major vectors of the parasite that causes onchocerciasis in Africa and Yemen, with other vector species involved in a few localized areas of Africa and in the Neotropics. Although the life cycle of these blackflies is linked to fast-flowing rivers, they can travel long distances (up to at least 500 km), calling into question how transmission zones are defined. Knowledge of the short- and long-range dispersal of these vectors could inform where control interventions and monitoring are necessary if targets for onchocerciasis elimination are to be met. Yet, research on blackfly dispersal has been limited and fragmented over the last 70 years. Here, we review the literature on the dispersal of onchocerciasis vectors, and we show the need for further research to establish how far larvae can travel downstream; the extent to which adults invade transmission zones; and whether adults migrate in a series of successive short movements or in single long-distance shifts, or use both methods.

## 1. Introduction

The ability of insect vectors of disease to travel in search of hosts and to move over long distances to expand their geographical ranges in seasonal, short-term, or long-term shifts is an important factor influencing the epidemiology of vector-borne diseases. This is especially true of onchocerciasis, colloquially known as “river blindness”, which is spread in Central America, sub-Saharan Africa, and Yemen by members of the genus *Simulium* (Diptera: Simuliidae). These vectors concentrate breeding in highly oxygenated sections of rivers, including rapids, from which they can disperse anywhere from a few kilometers up to many hundreds of kilometers. The precise nature of their dispersal underlies fundamental assumptions about onchocerciasis transmission and, consequently, how to achieve—and verify—its elimination.

Onchocerciasis is caused by parasitic nematode worms, *Onchocerca volvulus*, that live subcutaneously in human hosts, where they may survive for up to 12 years. The adult worms reside in nodules and produce numerous microfilariae as offspring that are taken up by biting female blackflies, *Simulium* sp. Once within a fly, the microfilariae transform into first-, second-, and third-stage larvae, with the latter being infective upon entering a person during a fly’s bite.

The principal vectors in Africa are members of the *Simulium* (*Edwardsellum*) *damnosum* species complex, originally described on the basis of banding patterns in stained polytene chromosomes found in their salivary glands [1]. The members of this species complex differ in their habitat requirements (forest, savannah, or forest–savannah mosaics) [1] and vectorial abilities [2]. Therefore, the identities of the vectors occurring in a focus of the disease are important considerations to be taken into account when planning or monitoring control campaigns, whether by vector control with larvicides [3], mass drug administration (MDA) targeting the parasite, or combinations of both interventions. Such campaigns are increasingly planned based on the idea of transmission zones. A transmission zone was originally delineated by an expert committee of the African Programme for Onchocerciasis Control (APOC) as “a geographical area where transmission of *O. volvulus* occurs by locally breeding vectors, and which can be regarded as a natural ecological and epidemiological unit for interventions” [4]. Operational definitions include river basins or large sections of these where the river is the core of the disease-endemic area and infection levels decline with increasing distance from the river [5]. This definition places suitable riverine breeding sites for the *Simulium* vectors of onchocerciasis as the initial point of reference from which a transmission zone extends. The spread of a transmission zone from this nucleus is primarily determined by infection prevalence in the human population, which, in turn, is a reflection of the potential range and dispersal of blackflies following their emergence at riverine breeding sites.

In the WHO’s later operational definition of a transmission zone, the rule of thumb includes all communities within 20 km of a river or its tributaries that are productive of blackflies [6]. Therefore, understanding the ability of flies to move distances of up to 20 km, treated here as short-range dispersal, and whether they are nulliparous or parous and, if the latter, also infective, is relevant to interpreting the dynamics of infection rates within a transmission zone, one of the main discussion points in this review. Related to this is the issue of where blackflies are collected and for what purpose. A third issue concerns long-distance migration by blackflies and its epidemiological implications, plus how it affects the logistics of control operations, including whether it negates the value of the transmission zone concept [7]. Crosskey reviewed the literature on the flight and migration of blackflies in general [8], but here, we concentrate only on movements by species known to be vectors of onchocerciasis. As part of our review of long-distance movements, we will discuss the two contrasting hypotheses about how flies achieve long-distance dispersal. One suggestion is that it is by “saltatory movements” in successive steps, taking a comparatively long time, while the other hypothesis proposes that these insects are carried by the wind for long distances rapidly in “direct flights” [7]. It is probable that both types of movement occur.

The definition and understanding of what constitutes dispersal and migration events are often confused [9], and different authors have used these terms for various concepts. Throughout this review, we will use the term dispersal for short- to mid-range displacements, which encompass larval drifting and flights either in search of a host, i.e., appetitive flights, or in search of an oviposition site; long-range displacements will be encompassed under the term ”migration”, independently of whether this is a result of active flight or passive wind transportation.

## 2. Locations of Blackfly Capture Points

Where blackflies are collected and for what purpose have practical implications. In order to investigate short-range dispersal, it is necessary to have fly-catching points beside the river at the breeding site and at increasing distances away from the source. This has seldom been accomplished except in experiments designed specifically for this purpose (see below). Most data on blackfly population dynamics and infection rates are derived from flies caught beside river banks.

Some countries are now preparing to determine whether they can stop MDA with ivermectin, the microfilarial drug of choice. Entomological sampling provides key data to inform programmatic decisions on whether to stop MDA and begin post-treatment surveillance or not. The cessation of transmission within a transmission zone is to be confirmed by pool-screening 6000 *Simulium* for *O. volvulus* using the O-150 PCR test, from which all blackflies must be negative to meet the criteria set by the WHO for the purpose of stopping MDA [6]. These guidelines also highlight the need to prioritize research to define appropriate and standardized protocols for catching these flies. Where these flies are collected may materially influence the resulting prevalence of infected flies as determined by PCR and, therefore, the outcome of decisions on stopping MDA.

Certainly, blackfly collections close to breeding sites have the advantage of ensuring collections happen where adult flies are likely to be found, possibly in the numbers required to reach the number of specimens stipulated in the WHO guidelines. However, high numbers of vectors alone may not be sufficient to indicate elimination if the vector population from which they were sampled is not representative of the infection prevalence in the wider population. This may be the case in some instances due to the dispersal and distribution patterns of blackflies at various physiological stages [10]. On the one hand, onchocerciasis is often a focal disease, and it is widely assumed that communities closest to vector breeding sites are at greatest risk of exposure to bites and are, therefore. likely to have a greater incidence of infection [5]. This is reflected in the designation of first-line villages—those geographically closest to or within a 5 km radius of an active breeding site and, therefore, considered to be at greatest risk—in which both epidemiological and entomological surveillance typically takes place (see [11,12] for examples). On the other hand, onchocerciasis modeling frameworks tend to be parametrized on the basis that biting risk is homogenous across a transmission zone, in no small part because entomological surveillance usually takes place only at these breeding sites or first-line villages and not across the wider transmission zone.

## 3. Variation in Exposure to Vector Bites

The spatial overlap between vector and human populations and how this may influence the risk of exposure to bites is an important consideration. Variation in individual exposure to vector biting may be driven by different biological factors including age [13]; sex [14]; individual attractiveness to flies [15]; and social determinants such as occupation [16]. How biting risk varies spatially in relation to blackfly populations and their movements is less clear. Yet, this could be a crucial factor in understanding the effectiveness of interventions and when it is safe to stop them. In lymphatic filariasis, different combinations of vector:host ratios can result in the same disease prevalence because it indicates average exposure only, with parasites able to persist at low levels when there is considerable variation in individual exposure to bites [17]. In onchocerciasis, variability in exposure is projected to lead to the density-dependent establishment of the *O. volvulus* parasite, specifically in lower endemicity settings, which could, therefore, contribute to transmission rebound following the premature cessation of MDA [18].

Spatial variability in biting risk is, therefore, relevant to both the practical surveillance and effective modeling of eventual elimination. An understanding of fly movement, both appetitive and migratory, will be useful in determining the optimum location for xenomonitoring blackflies during routine and elimination surveillance and in parametrizing models to project elimination and plan successful programs to achieve it.

There have been suggestions that *S. damnosum* s.l. could travel 55 km in Uganda [19] and disperse at a similar distance in Cameroon [20], and the first comprehensive study was by Le Berre [10]. He showed that there were differences between flies in different habitats and that numbers and parous rates decreased with increasing distances from breeding sites. At Tiassale (forest, Côte d’Ivoire), he found 1800 biting females/day/person—decreasing to 416 at 20 km away, 85 at 35 km, and 16 at 41 km of distance—and reduced dispersion in dry seasons. In the savannah, he found that flies dispersed radially from breeding sites in the wet season but linearly along riversides in the dry season. In Sudan Savannah, there was only linear dispersion along water courses in both seasons [10,21].

Regarding parous rates, Le Berre [10] found in Sudan Savannah that a parous rate of 61% at the riverside decreased to 41% 6 km away. In Guinea Savannah, the decrease was from 47 to 35.5% at 8 km away, and in the forest, only a marginal difference of 2% was noted between 7 and 41 km of distance. Similar results were found by Duke [22], Renz and Wenk [23], and Renz [24], who also found decreasing parous rates away from the river in Sudan Savannah (see Figure 2 in [23], from which quantitative data on both numbers caught and parous rates could be extracted and used for modeling purposes).

The above statements do not, however, reflect the full story. For instance, although Séchan [25] found lower parous rates away from breeding sites in 1979, the previous year, he found 88% parous rates 12 km away from a breeding site in Sudan Savannah. Furthermore, Garms and Walsh pointed out that in reinvasion studies (see below for further details on these), no nulliparous flies were ever found more than 25 km from breeding sites and that long-distance migrants were mostly old and included infective females [26]. Thus, it is likely that there is a difference between declining parous rates among *dispersing* populations and increasing parous rates among populations of long-distance (up to 500 km) *migrants*.

Duke found that in a Cameroonian Sudan Savannah zone, both parous and nulliparous *S. damnosum* dispersed inland and away from their breeding sites to the same distances, but the parous flies mostly stayed close to or along the banks of where they emerged, leading to a higher number of infective flies at breeding sites [22]. When compared with areas 1–3 km inland, this was estimated to increase transmission potential on the riverbank by a factor of two- to ten-fold. A similar pattern was found in Guinea Savannah. However, in a forest zone, the opposite was found, with a greater proportion of infective parous flies further inland away from the riverbanks, with nulliparous flies staying closer to the rivers.

De Sole et al. surveyed first- to fifth-line villages in Guinea (River Milo), Senegal (River Gambia), and Mali (River Niger) [27]. Only the Milo focus vectors (*S. sirbanum* with *S. soubrense* in the south) followed the expected pattern of first-line villages, having the highest infection levels, and these decreased with the increasing distance of the villages from breeding sites. In contrast, along the River Gambia (vector *S. sirbanum*), the villages with the highest endemicity were clustered closely along rivers, no more than 3 km from breeding sites, and were relatively few given the many breeding sites; those on tributaries seemed to be unimportant. Finally, at the Tienfala focus on the River Niger (vector *S. sirbanum*), several villages with high-intensity infection were second-line villages over 10 km from breeding sites, and severely affected villages with a prevalence over 60% were only found to the north of the river, with no obvious explanation for these observations.

In a Nigerian savannah mosaic habitat with a relict gallery forest along the river banks, Ozumba et al. caught unidentified members of the *S. damnosum* complex 0.02 km from the banks of the Oji River and at two sites further away [28]. They found averages of 3.6, 1.1, and 0.2 flies per man/hour at the river and 6.7 and 11.4 km away, respectively. A recent analysis of data collected under the WHO Onchocerciasis Control Programme (OCP) also reported numbers of *S. damnosum* s.l. (mostly *S. damnosum* s.str., but with some *S. squamosum* and some Beffa form of *S. soubrense*) declining with increasing distances up to 10 km away from a source river in a savannah habitat in Togo [29]. The authors also noted that the parous rate decreased with distance from the river, implying that nulliparous flies disperse further than parous flies and that some flies were infective up to 10 km from the river.

The annual biting rate (ABR) and annual transmission potential (ATP) were reported for transects of between 4 and 32 km from breeding sites around the Mbam and Sanaga rivers in Cameroon [30]. The results were mixed, with ABR and ATP broadly decreasing with distance from breeding sites at Mbandjock (up to 6 km) and Ngoro (up to 23 km), while at Song-Loulou (up to 4.3 km) and Bokito (up to 32 km), the opposite was found, and both ABR and ATP increased substantially at the furthest distances from breeding sites.

The community microfilarial load (CMFL) was found to decrease with distance from the river at the Mbam in Cameroon, an area of forest–savannah mosaic [31]. However, it should be noted that these results concern the CMFL rather than individual microfilarial prevalence, which did not vary much between villages (between 85.7 and 92%) located 6–8 km from the river. This is explained by strongly non-linear relationships between microfilarial prevalence and intensity and is a common issue because many papers do not report the intensity (CMFL), only prevalence.

## 4. Short-Range Dispersal by Blackfly Vectors of Onchocerciasis

In this section, we discuss evidence on the phenomenon of downstream larval dispersion, movements in relation to mating habits, and evidence of adult dispersion from mark–recapture experiments before considering long-range dispersal by blackfly vectors of onchocerciasis in a later section.

### 4.1. Larval Dispersion

Apart from familiar aerial movements by adults, blackflies can also disperse as immatures since larvae are capable of detaching from their aquatic supports, drifting downstream and then re-attaching to new supports after emitting silk threads as anchors. Crosskey reviewed drifting by blackfly larvae, which may be stimulated by some adverse events, such as a spate or the arrival, from upstream, of a pesticide, or can be voluntary as a behavioral means of finding new, possibly advantageous sites [8]. In both cases, there is a risk that the insects will be unable to find a new attachment site, especially if the river current is very strong. There is evidence that the dispersal rate of neonate blackflies from egg masses is density-dependent, more so at fast current speeds and when food is scarce [32].

Detachment in many species, including larvae of the *S. damnosum* complex [33], usually begins at dusk and continues as a nocturnal activity. The majority of larvae in the drift are early instars, with mature larvae apparently needing current speeds of more than 2 m s^−1^ to detach. The important aspect of drifting—as far as this review is concerned, the distances traveled by larvae—is enigmatic and lacking for the *S. damnosum* complex. Movements of up to 474 m have been proved by labeling larvae with radiophosphorus [34], but Russian authors have suggested much longer distances, with Patrusheva arguing that *S. transiens* can move at least 25 km and possibly 250 km downstream [35].

In parts of East Africa, onchocerciasis is transmitted by blackflies whose larvae and pupae are phoretic on crabs. It is unlikely that the crabs will travel far, and being attached to a large crustacean may hinder any propensity to larval drift. The adults are not known to disperse far, with Mpagi et al. noting maximum distances traveled by female *S. neavei* of 4 km [36].

### 4.2. Movements in Relation to Mating Habits

It is thought that female blackflies remain in the surroundings of their natal river for approximately 24 h after emergence [37]. Mating generally takes place near the emergence site during this post-emergence period [38]. The females then disperse away from the river surroundings to where they will have higher chances of finding a host [37]. This initial dispersion could be visually guided, as unfed flies have an aversion to highly reflective surfaces such as water bodies [39]. It is unclear at what time of the day this dispersal flight takes place, as some authors have suggested that it is likely to be during normal diurnal peaks of activities [37], although there are reports of actively flying blood-seeking blackflies that have been collected in the evening and up to 2.5 h after sunset [40].

### 4.3. Evidence for Adult Dispersal of Onchocerciasis Vectors, Mainly from Mark–Recapture Experiments

The first indications that vectors of onchocerciasis travel substantial distances from their natal areas were provided by mark–recapture experiments. Dalmat stained and released 19,580 females of *Simulium* spp. in Guatemala during a 94-day-long experiment [41]. Stained flies (9 *S. ochraceum*, 8 *S. metallicum*, and 4 *S. callidum*) were recovered at sites in all directions, suggesting that the direction of the wind was not influential. The flies were found between 2.1 miles (3.4 km) and 7.4 miles (11.9 km) away from the release site. One *S. metallicum* was re-caught 3.8 miles (6.1 km) away the day after it was released, suggesting a very fast flight, and Dalmat speculated that this meant that the flies could “travel great distances”. The experiment was repeated with similar results when the maximum distance flown, estimated on the assumption that a straight flight was involved, was 9.7 miles (15.6 km) [42]. In a third experiment, it was shown that flies infected with *O. volvulus* could travel at least 2.9 miles (4.7 km) [43].

There have been several mark–recapture studies of West African members of the *S. damnosum* complex. After marking and releasing 3886 flies likely to have been *S. sirbanum*, Crisp recovered one 12.9 km away from the release point [44]. Noamesi marked 19,648 flies, and the average distance traveled by those recovered was 95 km, with a maximum distance moved of 183 km [45]. After marking 33,000 flies, the maximum distance at which one was re-caught was 5 km in one study [46] and 17 km in a second study involving 62,878 flies, probably a mixture of S. *damnosum* s.str. and *S. sirbanum*, on the White Volta River in Ghana [47]. A recovery rate of 1.2% at the marking site was reported by Disney after marking 2300 flies in a forest zone in Cameroon [48]. Also working in the forests of Cameroon, Thompson monitored the numbers of marked flies recovered at different distances from the marking site, thereby measuring the decline in numbers at increasing distances from their origin [49]. Six flies were recovered at the maximum distance of 79 km, with one traveling this far in a day.

## 5. Long-Range Dispersal by Blackfly Vectors of Onchocerciasis

The possibility that blackflies could travel very long distances was suggested before it was demonstrated. Dalmat proposed that *S. metallicum* could traverse 150 km [41], Hocking showed that *S. venustum* and *S.vittatum* had the physiological attributes to travel non-stop for 100 km [50], Fredeen reckoned that *S. arcticum* could move 220 km [51,52], and Baranov thought that *S. columbaschensis* could travel for 250 km [53]. Le Berre [10] and Hausermann [46] suggested that *S. damnosum* s.l. could travel between 20 and 40 km. However, after *S. damnosum* s.l. was eliminated from the Nile at Jinja in Uganda, flies re-colonized there after a few years, apparently from sources at least 120 km away [54]. Wellington [55], quoted by Le Berre [56], wrote of American blackflies that, “Occasionally, large numbers of Simuliids invade a formerly untroubled area”, but it was only with the advent of the WHO OCP that the extent of migrations by *S. damnosum* s.l. was elucidated by experimental treatments following the occurrence of reinvasions at sites next to rivers treated with insecticides.

The OCP was a massive control operation, the initial aim of which was to eliminate onchocerciasis in the Volta River basin of West Africa by treating the riverine breeding sites of *S. damnosum* s.l. with weekly applications of larvicidal insecticides in 11 different countries [3]. The aim was to prevent onchocerciasis transmission for longer than the then-estimated 14-year maximum lifespan of the adult *O. volvulus* worms, thereby eliminating the disease as a public health problem. The OCP was established in 1974, began insecticidal treatments in 1975, and finished its main spraying operations in 2002, having supplemented its activities with the mass distribution of the microfilaricidal drug ivermectin starting in the late 1980s. The main aim of eliminating onchocerciasis as a public health problem was achieved [57].

The reinvasion phenomenon affected the OCP in four main ways: (1) movements that occurred during wet seasons when flies traveled from sources in the southwest of the original OCP area toward the northeast; (2) movements that occurred during wet seasons when flies traveled from sources in the southwest of the eastern extension of the OCP area toward the northeast; (3) movements at the ends of dry seasons with flies traveling back toward the southwest from the northeast; and (4) movements of flies resistant to the insecticide of choice (the organophosphate temephos).

### 5.1. Blackfly Migrations from the Southwest of the Original OCP Area toward the Northeast

Reinvasions in the west of the original (Phase I) area of the OCP were noticed as soon as the first season of operational spraying in 1975 began when unexpectedly high numbers of flies were caught along the Bandama and Leraba rivers in Côte d’Ivoire [56,58,59], even though no or very few larvae were still present in the treated sections of these rivers. As meteorological studies suggested that the arrivals of flies coincided with zones of wind convergence [60,61] and the prevailing winds were from the southwest to the northeast, it was surmised that wind-borne movements associated with the northward progression of the Intertropical Convergence Zone (ITCZ) were involved. The ITCZ is where winds converge creating a zone where rain is likely to fall and, therefore, to the benefit of the insects caught up in it [62], especially for those dependent on river flow. The reinvasion phenomenon recurred in 1976 when most (97–100%) of the flies caught within the controlled zone were parous, and many (5–15% on the Leraba) were infected with *O. volvulus* larvae. As the areas involved were sparsely populated or deserted, these findings added to the evidence that reinvasions were involved and that the parasite was reinvading too. Reared larvae from eggs obtained from reinvading flies were found to belong to the two savannah forms of *S. damnosum* s.l., *S. damnosum* s.str. and *S. sirbanum* [56].

Extensive research between 1975 and 1978 involving experimental treatments of potential sources of these flies confirmed that *S. damnosum* s.str. and *S. sirbanum* were reinvading and regularly traveling as far as 300 km and into Burkina Faso but occasionally also moving as far as 500 km [26,63,64]. It is important to emphasize that the numbers of flies involved were not trivial, with maximum numbers caught per day in excess of 380 and maximum monthly biting rates (MBRs) for the period of April to August of more than 18,000 [63]. An alternative approach to investigating the reinvasions was provided by Johnson et al., who analyzed the quantities and timings of flies appearing at different sites [65]. They noticed that the flies appeared in waves, with lags between their appearances increasing with increasing distances from potential sources. Analyses showed that there were statistically significant lags averaging one day per every 10–30 km away from the southwest OCP border in 1977 and one day per 7–35 km in 1978. These results suggested that the flies were not traveling in single extensive movements but rather were slowly traversing toward the furthest points within the OCP during a period of 31–48 days. This conclusion was consistent with estimates based on analyses of pteridine concentrations showing that female *S. sirbanum* invading Mali could live for an average of 29 days, with one estimated to have survived for 67 days [66]. Subsequently, Johnson and Johnson used a computer modeling approach to test the hypothesis that a batch of emerging flies migrates downwind together, with some laggards [67]. The models with the smallest proportions of laggards fit the data best. Further evidence for this saltatory type of migration was supported by laboratory experiments using flight mills, which showed that the maximum distance that a tethered female *S. squamosum* could fly was 5.25 km, with similar results for females of the Beffa form of *S. soubrense* (5.0 km) and *S. sirbanum* (5.27 km) [68].

When the OCP area expanded further west, similar reinvasions occurred into controlled parts of Guinea and western Mali [69,70,71], which were eventually curbed by treating rivers in Sierra Leone, some of which were more than 500 km away from the reinvaded sites [72]. In this study, it was found that there were waves of flies appearing at intervals at sites successively further away, as described by Johnson et al. for the original reinvasion studies [65].

### 5.2. Blackfly Migrations from the Southwest of the Eastern Extension of the OCP toward the Northeast of the Original OCP Area

In the east of the OCP, reinvasions were reported in Togo and Benin in 1979. In Benin, the flies reaching 200 km into the controlled zone were identified as *S. damnosum* s.str. and *S. sirbanum*, as in the west, with substantial numbers (maxima of >1200 per day) at sites on the Alibori and Bouli Rivers [73]. Such flies probably came from the Ouémé River to the southwest, given the directions of the winds at the time. In Togo, the situation differed, as many of the invading flies were *S. squamosum*, probably originating in the highlands along the Togo–Ghana border. These assumptions were confirmed by experimental treatments in 1980 and 1981 that showed that *S. squamosum* was indeed traveling 150 km northward and northeastward [74].

The movements of *S. squamosum* provided the first evidence that species other than the savannah forms (*S. damnosum* s.str. and *S. sirbanum*) of the *S. damnosum* species complex could travel far. Although often found in a variety of habitats, including savannah, *S. squamosum* is primarily an inhabitant of well-wooded highland areas. While it is generally assumed that forest species do not travel as far as savannah species, the now-extinct Djodji form of *S. sanctipauli,* a forest species, regularly expanded its range as far as 230 km from its dry season quarters in southwest Togo and southeast Ghana in gradual movements as wet seasons progressed [75]. A similar wet season expansion of the geographical range has also been noted for the forest-dwelling *S. yahense* [76], albeit over a much more restricted area delineated by the presence of thick forest.

### 5.3. Movements at the Ends of Dry Seasons with Flies Traveling Back toward the Southwest from the Northeast

There is a lack of clear-cut evidence that there is a regular return migration by flies at the end of the wet season, as the rivers that they populate during the wet season dry out, but there is evidence that this does occur sometimes. In 1988, a savannah form, *S. sirbanum*, suddenly appeared in southern Sierra Leone in forest areas [77], apparently in a southeastward movement associated with the return of the ITCZ. Other evidence from outside the OCP is provided by similar appearances of savannah forms in forested areas of Liberia [78] and Ghana [79]. In addition, cytotaxonomic markers studied by Boakye et al. revealed that savannah species only appeared in the lower reaches of the Comoe River in the dry season before being entirely replaced by forest species in the wet season [80], indicative of incursions from the north of savannah forms in the dry season.

### 5.4. Movements of Flies Resistant to the Insecticide of Choice (the Organophosphate Temephos)

The OCP began by using the organophosphate temephos as its larvicide of choice. However, in 1980, resistance to the pesticide emerged [81]. As it was crucial to know which rivers harbored resistant larvae, cytotaxonomic identifications were used to check which species were present, both where and when. Furthermore, as resistant larvae of *S. sanctipauli* s.l. carried a marker (inversion IIL-A), it was possible to track the movement of resistant populations. Meredith et al. used this technique to show that resistant insects, forest species, had moved from Côte d’Ivoire to Ghana [82].

## 6. Discussion

The results of studies on the short-range dispersal of *Simulium damnosum* s.l. females have implications for monitoring and evaluating onchocerciasis control programs. This is particularly relevant in the choice of blackfly monitoring sites and for epidemiological interpretations of riverside catches without supplementary catches away from rivers. For instance, Bockarie et al. [83] concluded from an investigation in Sierra Leone that “…classical riverside monitoring sites do not represent high risk areas for transmission of onchocerciasis in a forest village sited well away from the main *S. damnosum* s.l. breeding sites. The highest risk areas are in open farmland”.

In general, fly numbers and parous rates decrease with increasing distances away from breeding sites for dispersing flies, whereas the parous rates of migrating flies increase with increasing distances away from their rivers of origin. Current approaches to planning and monitoring onchocerciasis control programs tend to ignore migrant flies, and they do not feature in definitions of transmission zones. However, an examination of the results of reinvasion studies shows that the number of flies involved in migrations is very high and requires more serious consideration. In the late 1970s, reinvaded sites in Burkina Faso experienced average numbers of flies per man per day in the hundreds, with maxima in excess of 300 [63]. Such maxima in Togo regularly exceeded 400, and in Benin, they reached 1300 [73]. Assuming that such migrations evolved as an annual means by which rivers that dry out in the wet seasons for up to four months a year are re-populated as the rainy season progresses, there is no reason to suppose that, although unnoticed in the absence of larvicide operations, they are not still of considerable importance throughout savannah zones.

Our review has illustrated that further research is needed to establish how far the larvae of *S. damnosum* s.l. can travel downstream; the extent to which transmission zones are invaded by adults from elsewhere; and whether the migrations by adults take place in a saltatory manner, in direct flights, or by both such means. Incorporating answers to these questions with the existing body of knowledge will contribute to understanding issues such as how heterogeneity in individual exposure to blackfly bites may influence density-dependent parasite establishment in human populations [18]. Meanwhile, new blackfly sampling tools may also enable more intensive vector sampling at locations other than at known riverine breeding sites, thereby improving the spatial (and temporal) resolution of data on vector biting rates that lead to human exposure to parasites [84]. The movement of vectors both between and within communities at risk of exposure will need to be taken into consideration when delineating transmission zones on the pathway toward elimination.

## Data Availability

No new data were created or analyzed in this study. Data sharing is not applicable to this article.
